# Efficacy of Cannabinoids in Improving Nutritional Outcomes in Chemotherapy Patients: A Scoping Review

**DOI:** 10.7759/cureus.114757

**Published:** 2026-08-18

**Authors:** Omar Suleiman, Hrithik Saride, Kaila Gagne, Xenia Balulescu, Natalie Elazar, Jameel Kara, Melannie Ribeiro, Rebecca Riekki, Emily Rodman, Johnathan Wang, Stephanie N Petrosky

**Affiliations:** 1 Nutrition, Nova Southeastern University Dr. Kiran C. Patel College of Osteopathic Medicine, Clearwater, USA; 2 Nutrition, Nova Southeastern University Dr. Kiran C. Patel College of Osteopathic Medicine, Fort Lauderdale, USA

**Keywords:** appetite, cancer-cachexia, energy-balance, nausea, thc/cbd

## Abstract

Herbal cannabis has been utilized for thousands of years for its medicinal properties, including cardiovascular regulation, reproductive benefits, modulation of inflammation, and pain relief. Cannabinoids have also emerged as a therapeutic option for managing chemotherapy-related side effects, such as nausea, vomiting, weight loss, and loss of appetite. Understanding the unique responses of patients to chemotherapy and cannabinoids with respect to nutritional outcomes can refine oncological treatment protocols and significantly improve patients' quality of life. However, a preliminary search of MEDLINE and the Cochrane Database of Systematic Reviews found no existing or ongoing systematic or scoping reviews on the use of cannabinoids for key nutritional indices such as appetite, weight, and side effect management in patients undergoing chemotherapy. This scoping review aimed to assess the relationship between cannabinoid use and the reduction of side effects in cancer patients undergoing treatment, with the aim of identifying a positive association with improved dietary intake and thus nutritional status. Nine peer-reviewed original research publications met the critical appraisal criteria for inclusion. Data relating to weight loss, appetite, nausea, vomiting, and reported quality of life were analyzed. The included studies evaluated several cannabinoid interventions. Two small studies reported improvements in appetite, with one also demonstrating significant weight gain, whereas most studies found no significant differences in these nutritional outcomes compared with placebo or standard treatments. Limitations included small sample sizes and high dropout rates. This review highlights the complex and variable role of cannabinoids in patients undergoing chemotherapy. Overall, the evidence for improving nutritional outcomes remains inconsistent, underscoring the need for more rigorous research to determine the optimal patient population, dosing regimens, and long-term safety of cannabinoids. Clinicians should continue to monitor emerging evidence and consider cannabinoid therapy on an individualized basis when managing chemotherapy-related symptoms.

## Introduction and background

Herbal cannabis has been utilized for thousands of years for its medicinal properties. Cannabinoids are derived from the plant *Cannabis sativa*, which consists of over 500 known substances [1]. Cannabinoids are typically classified into three primary categories: phytocannabinoids, which are cannabinoids derived from plants; endocannabinoids, which are naturally occurring compounds in animals that interact with the same receptors as certain phytocannabinoids; and synthetic cannabimimetics, which are artificially created compounds that may or may not share structural similarities with phytocannabinoids but still display agonistic effects on cannabinoid receptors [2]. The two most notable phytocannabinoids are Δ-9-tetrahydrocannabinol (THC), which has psychoactive properties, and cannabidiol (CBD), which is nonpsychoactive [3]. These substances act upon the intrinsic human endocannabinoid system (ECS), consisting of cannabinoid neurotransmitters and endocannabinoid target receptors in multiple organs, ranging from those found in the central and peripheral nervous systems to the immune system [4]. The primary components of the ECS are the endocannabinoid ligands anandamide (AEA) and 2-arachidonoylglycerol (2-AG), which work in conjunction with G-protein-coupled receptors CB1 and CB2 to regulate physiological functions such as metabolism, cardiovascular control, reproduction, inflammation, immune system activity, and pain relief [5]. The Δ-9-tetrahydrocannabinol phytocannabinoid acts as an agonist of both CB1 and CB2 receptors. In animal studies, stimulation of CB1 by this compound has been linked to reduced locomotor activity, hypothermia, catalepsy, decreased pain sensitivity, and increased appetite. Activation of CB2, on the other hand, is primarily associated with pain relief and anti-inflammatory effects. However, CB2 displays less affinity for THC than CBD [6,7].

Chemotherapy remains a cornerstone of cancer treatment; however, it is frequently associated with significant adverse effects, including nausea, vomiting, anorexia, and cancer-related cachexia, a multifactorial syndrome characterized by involuntary weight loss, muscle wasting, and reduced appetite [8]. As people age, their risk of developing cancer rises, and physiological changes along with comorbid conditions make older adults more susceptible to severe adverse effects from chemotherapy [9]. Older individuals experience a decline in muscle mass and strength as they age, a condition known as sarcopenia. Alterations in body composition influence how chemotherapy is processed in the body, raising the risk of toxicity [9,10]. Older adults are at heightened nutritional risk; when compounded by sarcopenia, this predisposition may result in significant weight loss and poorer clinical outcomes during chemotherapy [11]. Polypharmacy is another prevalent issue among older individuals, raising the risk of drug-drug interactions that can lead to chemotherapy toxicity [10,12]. Due to these factors, older individuals require alternative treatment plans to manage the adverse effects of chemotherapy.

Cannabinoids have emerged as a therapeutic option for managing chemotherapy-related side effects such as nausea, vomiting, and cachexia, thereby enhancing the quality of life and health outcomes of cancer patients [13]. Dronabinol and nabilone have received FDA approval for addressing chemotherapy-induced refractory nausea and vomiting [14,15]. As previously mentioned, cannabinoids can bind to CB1 receptors and thereby suppress the emetic reflex by blocking the release of excitatory transmitters [7,14]. Additionally, cachexia is a prevalent complication in patients with cancer, affecting approximately 50-80% of cancer patients and contributing to at least 25% of cancer deaths [16]. Cannabinoids have been shown to increase appetite, which can lead to increased food intake and subsequent weight gain in patients undergoing chemotherapy [17-19]. While cannabinoids have the potential to address other side effects of chemotherapy, this review will focus solely on nausea, vomiting, weight loss, and loss of appetite. Ultimately, cannabinoids offer several potential benefits for managing chemotherapy side effects, thereby enhancing the overall quality of life and reducing the potential complications of malnutrition for patients undergoing treatment.

Objective and review question

This scoping review aimed to map the extent and type of available evidence on the relationship between cannabinoids, appetite changes, weight gain, and the reduction of side effects in cancer patients undergoing chemotherapy while identifying gaps in the existing literature. A preliminary search of MEDLINE and the Cochrane Database of Systematic Reviews did not identify any existing or ongoing systematic or scoping reviews evaluating the use of cannabinoids for key nutritional outcomes, including appetite, weight, and management of treatment-related side effects in patients undergoing chemotherapy. Current literature highlights the benefits of cannabinoids for managing chemotherapy side effects across all age groups but fails to address the unique responses of patients to chemotherapy and cannabinoids with respect to nutritional outcomes. Enhancing physicians’ understanding of how older individuals respond to chemotherapy and cannabinoids can refine oncological treatment protocols and significantly improve patients' quality of life. The objective of this scoping review was to address the question: *What are the effects of cannabinoids on subjective appetite scores, weight gain, and chemotherapy-induced side effects in patients undergoing chemotherapy?*

## Review

Methods 

*Search Strategy Details* 

The research question was developed using the Population, Concept, and Context (PCC) framework. In this framework, the “P” represents patients undergoing chemotherapy, the first “C” refers to the effects of cannabinoids on self-reported appetite, weight gain, chemotherapy side effects (such as nausea and vomiting), and nutritional outcomes in cancer patients, and the second “C” denotes the inclusion of peer-reviewed articles written or translated into English. The research question was: “What are the effects of cannabinoids on subjective appetite scores, weight gain, and chemotherapy-induced side effects in patients undergoing chemotherapy?” Three databases (Embase, Ovid MEDLINE, and Web of Science) were searched in September 2024 using the following search terms: “cannabinoid” AND “cancer chemotherapy” AND (“appetite” OR “weight gain”) in the article title, abstract, or keywords. The descriptors were combined in different ways, aiming to broaden the searches. A complete map of the search methodology is provided in the Appendices.

Inclusion/Exclusion Criteria

Included articles were peer-reviewed and either written or translated into English. Studies evaluated the effects of cannabinoids on appetite (self-reported), weight gain, chemotherapy-related side effects (such as nausea and vomiting), and nutritional outcomes in cancer patients, both inpatient and outpatient. Due to limited availability, the analysis was expanded to include studies conducted outside the United States, recognizing that other countries have different regulations regarding cannabinoid use. Additionally, no publication date restrictions were set for the articles used.

This scoping review considered both experimental and quasi-experimental study designs, including randomized controlled trials, nonrandomized controlled trials, analytical observational studies, and case-control studies. Articles that presented survey-type data were prioritized, as these provided patient-reported outcomes along with statistical evidence on whether cannabinoids had a positive or negative impact on chemotherapy side effects. Case studies were included to assess the long-term benefits and potential risks of cannabinoid use during chemotherapy treatment.

Excluded publications were abstracts, editorial articles, not peer-reviewed, and not available in English. Only articles using human subjects met the criteria. In vitro and animal studies were excluded because the data provided would not directly apply. Following the application of these criteria, a total of 15 full-text articles were deemed eligible for this review. 

Study Selection

The initial search yielded a total of 589 articles. Of these, 76 duplicates were removed. Following the search, all identified citations were collated and uploaded to EndNote and Rayyan. Two independent researchers examined the titles and abstracts of the remaining sample, performing abstract screening and reaching a consensus on the articles for further consideration (n = 272) (Tier I). From there, articles were further screened, and discrepancies were resolved by a third reviewer. Twenty-seven articles were selected for full-text review.

**Figure 1:**
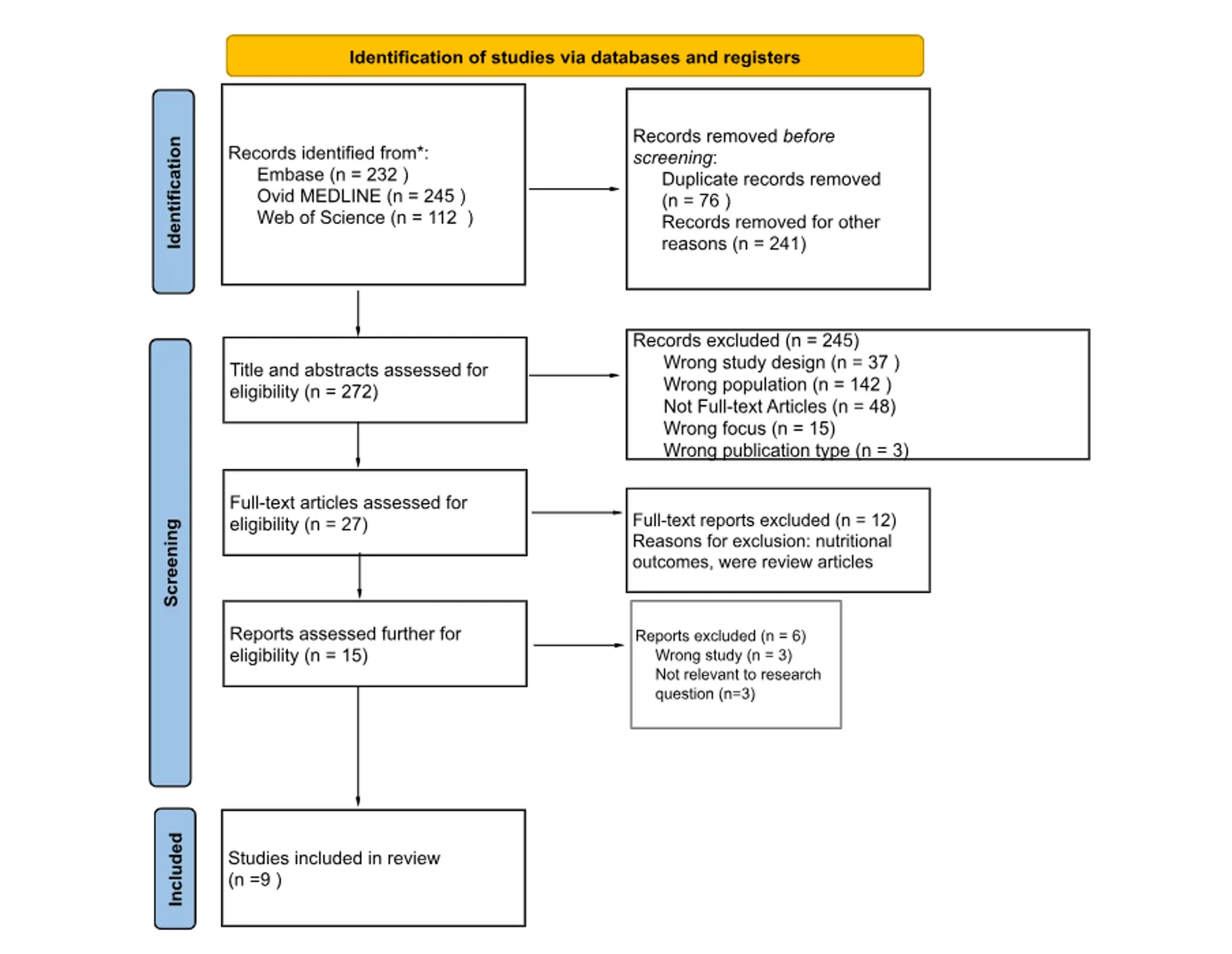
Preferred PRISMA-ScR flow diagram for the search and selection process PRISMA-ScR: Preferred Reporting Items for Systematic Reviews and Meta-Analyses extension for Scoping Reviews.

Two independent researchers evaluated the remaining pool for viability (Tier II). In cases of discordance, the article was presented to a third independent reviewer to reach consensus. As a result, 12 articles were excluded as they were review articles or did not include nutritional outcomes (Figure 1).

Following full-text review, 15 studies remained for further eligibility assessment. Six studies were subsequently excluded because they did not sufficiently address the review question or meet the predefined eligibility criteria, leaving nine studies for inclusion in the review. The included studies were then critically appraised using the Joanna Briggs Institute (JBI) critical appraisal tools in accordance with JBI methodology for scoping reviews [20]. Individual sources of evidence were assessed for methodological quality and potential risk of bias.

Data Collection

Data extraction was conducted by two independent researchers using Microsoft Excel (Microsoft Corporation, Redmond, Washington) and Rayyan (Rayyan Systems, Inc., Cambridge, Massachusetts) to manage records. The extracted data included specific details on the effects of cannabinoids on appetite improvement (self-reported), weight gain, chemotherapy side effects (nausea/vomiting), and nutritional gain among chemotherapy patients in the United States. Table 1 presents information related to the study design, purpose of the study, population, sample size, methods, key findings associated with the review question, and limitations of the study. As this was a scoping review, no meta-analysis was performed, and findings were synthesized narratively.

**Table 1:** Summary of the included studies

Author/Title	Study Design	Purpose	Study Population/Size	Methods	Key Findings	Limitations
Pomeroy et al. 1986 [21]; Prospective randomized double blind trial of nabilone versus domperidone in the treatment of cytotoxic-induced emesis	Randomized double-blind trial	Assess the effectiveness of domperidone vs nabilone in reducing cytotoxic-induced emesis in patients receiving chemotherapy that is highly emetogenic and compare their side effect profiles	38 subjects of 21- to 66-year-old patients with advanced malignant disease receiving chemotherapy. Male and female	Patients were randomized to a domperidone or nabilone group and received two cycles of chemotherapy. Patients filled out a diary card, and investigators filled out a record form each day of chemotherapy treatment.	Summary: The nabilone group had a statistically significantly lower number of vomiting episodes in cycle 1 and the combo of cycle 1/2 compared to the domperidone group. There was not a statistically significant difference in nausea score, food intake score, and vomiting episodes for cycle 2 between the two treatment groups. However, the trends pointed toward nabilone being more effective. The nabilone group experienced more side effects like euphoria, dizziness, postural hypotension, etc. when compared to the domperidone group.	Small sample size. Short-term study. 7 patients only completed one cycle of treatment. 4 patients dropped out before the end of the study
Niiranen and Mattson 1985 [22]; A cross-over comparison of nabilone and prochlorperazine for emesis induced by cancer chemotherapy	Double-blind crossover study	Assess the effectiveness of nabilone vs phenothiazines (PCP) in the prophylaxis and treatment of emesis related to chemotherapy for cancer	32 subjects of 48- to 78-year-old patients with lung cancer receiving two courses of identical chemotherapy. Male and female	Subjects were blindly and randomly assigned to the nabilone or PCP treatment group. All participants received one capsule the night before, 1 hour before, and at 12-hour intervals until 24 hours after chemotherapy. Symptoms were assessed by the investigator and through a self-administered questionnaire.	Summary: The study found that the number of vomiting episodes was lower with nabilone compared to PCP. There was no difference in severity of nausea between the two treatment groups. Additionally, side effects were experienced with nabilone more compared to PCP (ex: vertigo, dry mouth, etc.). However, more patients preferred nabilone over PCP when on chemotherapy.	Small sample size. 8 subjects removed from the study. Short-term study
Niiranen and Mattson 1987 [23]; Antiemetic efficacy of nabilone and dexamethasone: a randomized study of patients with lung cancer receiving chemotherapy	Randomized, double-blind crossover	Assess whether the addition of dexamethasone to nabilone would improve antiemetic effects and/or reduce side effects	40 subjects of 44- to 79-year-old lung cancer patients of all types having a performance status of 60% or more on the Karnofsky scale staying on 2 consecutive cycles of the same chemotherapy regimen	Patients were randomly assigned to receive 3 fixed doses of nabilone + placebo or nabilone + high-dose dexamethasone during the first cycle of chemotherapy; identical placebo and dexamethasone were only given during the first dose of nabilone the evening before chemotherapy; the two study groups would go on to receive the alternate treatment regimen on the second cycle of chemotherapy; Symptoms were assessed by a combination of investigator assessment and patient questionnaire	No change in patient assessment of appetite across the two groups; The combination of nabilone + high-dose dexamethasone was demonstrated to have greater antiemetic effects and a lesser degree of hypotensive effects as compared to nabilone alone; overall degree of nausea was shown to be lower with nabilone + high-dose dexamethasone	Small study population; eight patients removed due to various reasons; short-term study; difficult to maintain standardization of nausea
Côté et al. 2016 [24]; Improving Quality of Life with nabilone during Radiotherapy Treatments for Head and Neck Cancers: A Randomized Double-Blind Placebo-Controlled Trial	Randomized Double-Blind Placebo- Controlled Trial	Assess the effectiveness of nabilone (a synthetic cannabinoid) at reducing side effects related to radiotherapy for head and neck cancer	56 subjects of 18- to 80-year-old patients with squamous cell carcinoma of the oral cavity to larynx being treated with radiotherapy. Patients were treated at Hôtel-Dieu de Québec hospital from May 2005 to August 2007. Male and Female	Patients were randomized to nabilone or placebo groups. Patients filled out the European Organisation for Research and Treatment of Cancer QLQ-C30 and EORTC QLQ-H&H35 before radiotherapy, each week during, and 4 weeks after radiotherapy. Additionally, each week prior to radiotherapy, the dose of treatment was increased by 1 pill until they reached a maximum of 4 pills a day.	Summary: Nabilone did not have a statistically significant effect on adverse effects associated with radiotherapy in head and neck cancers. There was no difference in pain, appetite, body weight, nausea, sleep, mood, and quality of life between the nabilone and placebo group	Small sample size from one location. 24 study participants dropped out before the end of the study
Bar-Sela et al. 2019 [25]; The Effects of Dosage-Controlled Cannabis Capsules on Cancer-Related Cachexia and Anorexia Syndrome in Advanced Cancer Patients: Pilot Study	open-label, nonrandomized	To assess whether cannabis capsules can lead to significant weight gain (≥10% from baseline) and improve appetite in patients suffering from Cancer-Related Cachexia and Anorexia Syndrome	24 Adults (>18 years of age) with advanced cancer diagnosed with cancer-related cachexia and anorexia syndrome, having sufficient functional status (Karnofsky Performance Status ≥ 50) and no substance abuse history	Participants received cannabis capsules containing tetrahydrocannabinol (THC) and cannabidiol (CBD). The initial dosage was 10 mg THC and 0.5 mg CBD twice daily. However, if side effects occurred, the dosage was subsequently reduced to 5 mg THC and 0.25 mg CBD twice daily. Patients were weighed at each physician visit.	Among the patients who completed the study, 50% achieved the primary endpoint of ≥10% weight gain, while the others maintained stable weights. Patients reported reduced appetite loss after treatment (p=0.05). Self-reports indicated improvements in appetite, mood, pain, and fatigue.	High dropout rate; self-reporting bias and non-standardization
Brisbois et al. 2011 [26]; Delta-9-tetrahydrocannabinol may palliate altered chemosensory perception in cancer patients: results of a randomized, double-blind, placebo-controlled pilot trial	Randomized, double-blind, placebo-controlled pilot study	Determine whether delta-9-tetrahydrocannabinol (THC) improves chemosensory perception, appetite, caloric intake, and quality of life (QOL) in cancer patients experiencing altered taste and smell	46 Adult patients with advanced cancer (defined as locally recurrent, locally advanced, or metastatic) of any site except the brain who had a score ≥2 (out of 16) on a scored Taste and Smell Survey	Participants received THC or placebo twice daily for 18 days, with the option to increase dosage based on tolerance. Participants completed self-reported outcomes through the Taste and Smell Survey, 3-day food record, appetite, and macronutrient preference assessments, QOL questionnaire, and an interview at baseline and posttreatment.	Patients reported improved taste and smell perception, enhanced food enjoyment, and increased appetite compared to placebo. Patients in the THC group also reported better sleep and relaxation, with no major adverse effects. Protein intake increased, but total caloric intake remained unchanged.	High dropout rate
Sahli et al. 2024 [27]; Experience with medical marijuana in a pediatric palliative care clinic: Case Report	Case Report	Assess the effectiveness of medical marijuana in managing refractory symptoms in pediatric patients receiving palliative care	Three patients from a pediatric palliative care clinic at a Midwestern pediatric hospital system. 11-year-old male with rhabdomyosarcoma, 11-year-old male with Lennox-Gastaut Syndrome, and a 15-year-old female with hypoxic brain injury and spastic quadriplegia	Case reports were chosen due to the fact that they represent the majority of symptoms for which medical marijuana is recommended to be used (e.g., refractory seizures, spasticity, and cancer-related symptoms). Each patient's regimen was a 1:1 THC:CBD oil-based tincture three times daily.	Summary: Of the subjects assessed, they found a 1:1 THC:CBD to be effective at alleviating symptoms associated with the three disorders evaluated. In the patient with rhabdomyosarcoma, there was reported improvement in nausea, appetite, body weight, anxiety, and pain. The patient with spastic quadriplegia reported improved use of her facial muscles, global tone, and anxiety. The patient with Lennox-Gastaut Syndrome reported improved mood, less aggressive behavior, and seizure reduction. Finally, all three patients reported a decrease in polypharmacy.	Small sample size. Limited generalizability of findings. Highlights very specific cases
Sallan et al. 1980 [28]; Antiemetics in patients receiving chemotherapy for cancer	Randomized, double-blind, crossover trial	Assess the effectiveness of Delta-9-tetrahydrocannabinol (THC) vs prochlorperazine in reducing emesis in patients receiving chemotherapy who are unresponsive to standard antiemetic therapy	84 subjects of 9- to 70-year-old patients with known neoplasms, receiving chemotherapy but resistant to standard antiemetic therapy. Male and female	Each patient completed three courses of chemotherapy. On each day the patient received antiemetic treatment one hour before chemotherapy, 3 hours, and 7 hours after chemotherapy. Over the three days, the patients randomly received two courses with one drug and one course with another drug (ex: THC/prochlorperazine/THC, prochlorperazine/THC/THC, etc.). Patients completed a self-administered questionnaire after the treatment.	Summary: The study found that THC had a greater effect as an antiemetic compared to prochlorperazine. Additionally, the patients who received both drugs preferred THC to prochlorperazine.	Small sample size. Specific patient populations (refractory to standard antiemetic therapy). Only 38 patients completed all 3 courses of treatment. 27 patients were removed from the study
Strasser et al. 2006 [29]; Comparison of orally administered cannabis extract and delta-9- tetrahydrocannabinol in treating patients with cancer-related anorexia-cachexia syndrome: A multicenter, phase III, randomized, double-blind, placebo-controlled clinical trial from the Cannabis-In-Cachexia-Study-Group	Randomized, double-blind, placebo-controlled pilot study	To assess the effects of cannabis extract (CE), delta-9-tetrahydrocannabinol (THC), and placebo (PL) on appetite and QOL in patients with cancer-related anorexia-cachexia syndrome	243 Adult patients with advanced cancer, CACS, weight loss (≥5% over 6 months), and Eastern Cooperative Oncology Group (ECOG) performance status (PS) ≥ 2	Participants were randomly assigned to a CE, delta-9-tetrahydrocannabinol (THC), and placebo (PL) group after 7 to 14 days of baseline assessment. Patients received a 2-week supply of capsules to take orally twice daily. This occurred for a 6-week period, during which QOL and appetite assessments were made at each 2-week follow-up.	No significant differences were noted between the three groups over the 6-week trial for the primary end points of appetite and QOL, for cannabinoid-related toxicity, or for secondary end points such as mood or nausea.	Lack of intra-patient dose escalation of CE

Results

Overview of Included Studies

Figure 1 shows the PRISMA (Preferred Reporting Items for Systematic Reviews and Meta-Analyses) flowchart, which outlines how studies were included and excluded from the review. This review featured nine original research publications, most of which were randomized, double-blind trials and took place outside the United States. Participant ages ranged from 9 to 79 years, with the majority being adults diagnosed with various cancer types and cancer-related conditions such as cancer-related cachexia and anorexia syndrome (CACS) and emesis secondary to treatment. Sample sizes ranged from 3 to 243 participants, though six studies had small cohorts and three studies had high dropout rates [21-28]. These studies featured cannabinoid interventions, such as nabilone, delta-9-tetrahydrocannabinol, and cannabis extract, mainly administered through oral capsules at fixed doses. Measured outcomes focused on symptom relief from chemotherapy and radiotherapy, including nausea, vomiting, appetite, weight loss, and overall quality of life.

Descriptive Summary/Data Synthesis

A structured critical appraisal of the included studies was conducted using JBI tools appropriate to each study design to characterize methodological quality and potential sources of bias. Table 2 summarizes selected methodological domains identified during the critical appraisal.

**Table 2:** Summary of study-wise critical appraisal using Joanna Briggs Institute (JBI) tools Critical appraisal was conducted using Joanna Briggs Institute (JBI) critical appraisal tools appropriate to study design. For randomized controlled trials, the JBI RCT checklist was used; for case series and case reports, the respective JBI tools were applied. In accordance with scoping review methodology, no studies were excluded based on quality assessment.

First Author (Year)	Study Design	JBI Checklist Used	Randomization	Allocation Concealment	Blinding	Follow-up Completeness	Outcome Measurement	Confounding Control
Pomeroy (1986) [21]	RCT	JBI RCT Checklist	Yes	Unclear	Yes	Yes	Yes	Yes
Niiranen (1985) [22]	RCT (crossover)	JBI RCT Checklist	Yes	Unclear	Unclear	Yes	Yes	Yes
Niiranen (1987) [23]	RCT	JBI RCT Checklist	Yes	Unclear	Unclear	Yes	Yes	Yes
Côté (2016) [24]	RCT	JBI RCT Checklist	Yes	Yes	Yes	Yes	Yes	Yes
Bar-Sela (2019) [25]	Pilot study (case series)	JBI Case Series Checklist	No	No	No	Partial	Yes	No
Brisbois (2011) [26]	Pilot RCT	JBI RCT Checklist	Yes	Yes	Yes	Yes	Yes	Partial
Sahli (2024) [27]	Case report	JBI Case Report Checklist	N/A	N/A	N/A	N/A	Yes	N/A
Sallan (1980) [28]	RCT	JBI RCT Checklist	Yes	Unclear	Yes	Yes	Yes	Yes
Strasser (2006) [29]	RCT	JBI RCT Checklist	Yes	Yes	Yes	Yes	Yes	Yes

Randomized controlled trials generally demonstrated moderate to high methodological quality; however, earlier studies frequently reported unclear allocation concealment and inconsistencies in blinding procedures. More recent trials demonstrated improved methodological rigor. Nonrandomized evidence, including pilot studies, case series, and case reports, demonstrated lower methodological quality overall due to inherent design limitations such as lack of control groups and small sample sizes but was retained to ensure comprehensive mapping of available evidence in accordance with scoping review methodology.

Overall, a trend emerges from studies showing cannabinoids, particularly nabilone, as effective in reducing chemotherapy-induced vomiting compared with standard antiemetics and placebos. Pomeroy et al. and Niiranen et al. [21,22] consistently demonstrated that nabilone significantly reduced the frequency of vomiting episodes more effectively than prochlorperazine or domperidone. However, the impact of cannabinoids specifically on nausea remains inconsistent across studies. For example, Niiranen et al. [23] found that combining nabilone with a steroid like dexamethasone further enhanced antiemetic effects, yet the overall effect on nausea was not as robust. Additionally, Cote et al. [24] showed that nabilone did not have a notable impact on adverse symptoms associated with radiotherapy, suggesting that its benefits may be more specific to chemotherapy-induced nausea and vomiting.

When examining appetite and weight loss, most studies reported no significant differences between cannabinoid and placebo groups. However, Bar-Sela et al. [25] reported that approximately half of participants experienced greater than 10% weight gain, while Brisbois et al. [26] observed improvements in taste and smell perception that were associated with improved appetite. These encouraging findings, however, were derived from small pilot studies with limited sample sizes and notable dropout rates and therefore should be interpreted with caution until confirmed in larger, well-designed clinical trials.

In the realm of quality of life, mood, fatigue, and pain, there was also a notable lack of consistency across studies. Some research, such as that by Brisbois et al. [26], indicated that THC use led to better sleep and relaxation, suggesting potential improvements in quality of life. Sahli et al. [27], on the other hand, highlighted the benefits of a 1:1 THC:CBD combination in pediatric patients, where it helped reduce polypharmacy and manage symptoms associated with conditions like rhabdomyosarcoma, Lennox-Gastaut syndrome, and spastic quadriplegia. These results point to potential advantages for specific symptom management, though the broader impacts on mood and fatigue remain more variable across studies.

Overall, cannabinoids demonstrated varying degrees of efficacy across different outcomes, with the clearest evidence supporting a benefit in managing chemotherapy-induced vomiting. However, much of this evidence is derived from older studies that compared cannabinoids with antiemetic regimens that are no longer considered standard of care. Consequently, the applicability of these findings to modern chemotherapy protocols remains uncertain and warrants further investigation.

Adverse Effects

A few of the studies reported more side effects among patients receiving cannabinoids than among those receiving the comparator antiemetic therapies. Common adverse effects included dizziness, euphoria, vertigo, postural hypotension, and dry mouth. Despite these adverse effects, patients in several studies expressed a preference for cannabinoids over the comparator antiemetics. However, these findings were largely derived from studies conducted more than three decades ago using antiemetic regimens that are no longer considered standard of care. As a result, patient preferences may differ in the context of contemporary antiemetic protocols and newer cannabinoid formulations [22,28].

Gaps in the Literature

In reviewing the research literature, several gaps were identified, particularly in the patient populations studied. Most of the research concentrated on patients with advanced cancer, often those who were resistant to other antiemetic treatments. This narrow focus limits the generalizability of the findings to the broader group of cancer patients experiencing nutritional problems secondary to chemotherapy.

Another factor limiting the applicability of the studies is their small sample sizes. A majority of the studies began with 20-50 subjects, but due to high dropout rates from side effect intolerance or cancer progression, the final sample sizes were further reduced. For instance, in Cote et al. [24], the initial group of 56 subjects decreased by 24 participants by the end of the study, which compromises the validity of the results. Moreover, future research should prioritize randomized, double-blind studies.

While studies like Sahli et al. [27] offer valuable insight into the potential effectiveness of cannabinoids as an antiemetic, anxiolytic, and appetite stimulant, their findings are based on three specific cases, making it difficult to generalize to a larger population. These limitations highlight the need for more diverse studies with larger sample sizes to enhance the reliability and applicability of the research findings.

Variation in Findings

A few studies [24,29] failed to demonstrate the effectiveness of cannabinoids in improving appetite, alleviating side effects of chemotherapy, and promoting weight gain. Strasser et al. [29] conducted a study on the use of cannabis extract versus delta-9-tetrahydrocannabinol for cancer-related anorexia-cachexia and found no significant difference in appetite between the treatment groups and the placebo group. Similarly, Cote et al. [24] investigated the use of nabilone (a synthetic cannabinoid) in improving the quality of life of patients undergoing radiotherapy. They concluded that nabilone did not significantly reduce the incidence of side effects associated with radiotherapy compared with the placebo group. This study, in particular, differed from the others in that patients were undergoing concurrent radiotherapy, whereas the others studied patients undergoing chemotherapy alone. Future research should aim to clarify the efficacy of cannabinoids in preventing adverse nutritional outcomes, such as weight loss and reduced appetite, in patients receiving chemotherapy.

Contextual Factors

Further investigation into factors that may influence an individual's metabolism of cannabinoids, as well as cannabinoid dosing, is needed. Pomeroy et al. [21] highlighted instances of dropout due to adverse effects of cannabinoids despite all subjects having received the same dosage. This discrepancy suggests that genetic and lifestyle differences may play a significant role in determining an individual's response to cannabinoids. Bar-Sela et al. [25] showed that six of the 17 participants discontinued the study due to cannabinoid-related adverse effects. Of these, 67% withdrew while receiving the 10 mg dose, whereas 33% discontinued during the 5 mg dose phase. Furthermore, the study employed a ratio of 9.5 mg THC to 0.5 mg CBD for the 10 mg cannabinoid dose and used 4.75 mg THC and 0.25 mg CBD for the 5 mg cannabinoid dose, suggesting that the dose, as well as the ratio of THC to CBD, may influence the rate of adverse effects from cannabinoids.

Discussion

Summary of Findings

Overall, the findings suggest that nabilone improves symptoms such as vomiting compared with other antiemetic treatments; however, the impact of cannabinoids on nausea, appetite, and quality of life is less consistent and shows variability in how cannabinoids interact with different symptoms and patient populations.

Vomiting and Nausea

Cannabinoids, particularly nabilone, demonstrate a decrease in the number of chemotherapy-induced vomiting episodes across multiple studies, such as Pomeroy et al. and Niiranen et al. [21,22]. These studies determined that nabilone was more effective in preventing vomiting than traditional antiemetic medications such as prochlorperazine or domperidone. Additionally, Niiranen et al. [23] showed increased efficacy of nabilone when used with adjunct steroid therapy such as dexamethasone. This suggests that cannabinoids may interact with the central nervous system, which plays a role in regulating nausea and vomiting. However, results regarding the improvement of nausea were inconsistent, as the studies failed to show a significant difference when comparing the cannabinoid and placebo groups. According to Cote et al. [24], there was no significant effect of nabilone on the adverse symptoms related to radiotherapy in cancer treatment. The variability in results may be associated with differences in study design, treatment dosages, or patient characteristics; this highlights the complexity of the use of cannabinoids in clinical practice. The results from the review are consistent with other existing studies.

Appetite and Weight Loss

Another area of interest is the impact of cannabinoids on appetite and weight loss, which are common side effects of chemotherapy. Most studies did not demonstrate significant improvements in appetite or weight, although a small number reported favorable findings. Bar-Sela et al. [25] observed that THC/CBD capsules were associated with greater than 10% weight gain in approximately half of participants, along with self-reported improvements in appetite. Similarly, Brisbois et al. [26] reported improved taste and smell perception, leading to enhanced food enjoyment and increased appetite among patients receiving THC compared with placebo. However, these findings were derived from small pilot studies with limited sample sizes and methodological limitations, including a nonrandomized case series design in Bar-Sela et al., and therefore should be interpreted with caution.

Although the variation in results suggests that not all patients benefit equally, this may be attributed to differences in patient genetic factors, type and stage of cancer, or possibly prior treatments. While cannabinoids show promise for appetite and weight loss management, they are not universally effective for all patients, and further research is needed to determine which factors predict a positive response.

Quality of Life, Mood, and Fatigue

Several studies outline self-reported improvements in quality of life, mood, fatigue, and pain among cancer patients undergoing chemotherapy with the use of cannabinoids. Brisbois et al. [26] demonstrated improved sleep and relaxation in the THC group; this aligns with prior studies that indicate the sedative effects of cannabinoids. Similarly, Sahli et al. [27] reported that a pediatric patient treated with a 1:1 THC:CBD ratio showed improved mood and decreased aggression. The findings suggest that there are benefits to cannabinoids that extend beyond the control of chemotherapy-induced side effects and may improve the overall course of cancer treatment. However, some studies failed to detect significant differences between cannabinoid and placebo groups. For example, Strasser et al. [29] reported no significant differences in reported quality of life between two cannabinoid groups and the placebo group over a six-week trial.

Fatigue, pain, and mood disturbances are common but complex symptoms associated with cancer and chemotherapy and therefore might not respond as reliably to cannabinoids as vomiting or nausea. These elements are self-reported, which adds the challenge of patient subjectivity to the conflicting findings in the study. Psychological and socioeconomic factors, such as individual coping strategies and established support systems, can also influence how a patient perceives the impact of cannabinoids as part of their treatment.

Adverse Effects

Cannabinoids were associated with a higher incidence of side effects, including dizziness, euphoria, vertigo, postural hypotension, and dry mouth, compared with standard antiemetics. Niiranen et al. and Sallan et al. [22,28] reported that patients preferred cannabinoids over traditional treatment options despite the adverse effects. These findings suggest that while cannabinoids are associated with adverse effects, the patients' perceived benefits may outweigh the risks.

The increased incidence of side effects warrants caution in their use, especially in vulnerable patient populations. Side effects of cannabinoids, such as dizziness and postural hypotension, may be more pronounced in older patients or those with comorbidities. This highlights the need for personalized care when prescribing cannabinoids and suggests that patient education is an important part of treatment.

Clinical Implications

Current evidence for the use of cannabinoids to improve nutritional outcomes in patients undergoing chemotherapy is limited and inconsistent. While a small number of studies reported improvements in appetite, weight, or related nutritional measures, most studies found no significant differences compared with placebo or standard treatments. Evidence supporting the management of chemotherapy-induced vomiting appears more consistent; however, much of this evidence is derived from older studies using antiemetic regimens that differ from current standards of care.

The variability in findings highlights the need for a personalized approach to cannabinoid therapy, as patient responses may differ according to cancer type, disease stage, treatment regimen, and individual characteristics. Although cannabinoids may provide symptomatic benefit for selected patients, their routine clinical use remains limited by inconsistent evidence, variable patient responses, adverse effects such as dizziness and postural hypotension, and regulatory challenges surrounding standardized formulations. Future research should focus on large, well-designed randomized controlled trials to identify the patients most likely to benefit and to establish standardized dosing strategies and treatment protocols.

Recommendation for Future Research

This area of research is inherently limited by high dropout rates and small sample sizes. Subsequent studies need to focus on conducting large, randomized, double-blind clinical trials to evaluate both the effectiveness and safety of cannabinoids for patients undergoing chemotherapy. Future research should investigate how genetic and metabolic differences affect individual responses to cannabinoids, as current studies show varying results in appetite stimulation and weight maintenance. In addition, long-term studies would add to the understanding of the effects of chronic cannabinoid use and the risk of dependence in susceptible populations such as older adults. Multimodal treatment approaches, specifically their interactions with standard antiemetic medications and appetite-stimulating drugs, should be explored. Further, pharmacokinetic studies alongside standardized dosing methods will play a vital role in enhancing therapeutic application. The growing legalization of cannabis throughout multiple regions necessitates enhanced real-world data collection and postmarketing surveillance studies to understand patient-reported outcomes and adverse effects in clinical settings.

Limitations

This review has several limitations. First, the search strategy was limited to peer-reviewed literature indexed in the selected databases and did not include gray literature or clinical trial registries, which may have introduced publication bias. Second, this scoping review was not prospectively registered. Third, the literature search was completed in September 2024 and was not updated prior to manuscript submission; therefore, studies published after the search date may not be represented in this review. Additionally, restricting the search to title, abstract, and keyword fields may have excluded relevant studies that did not explicitly report these terms, introducing the potential for selection bias. Finally, variation in study design, patient populations, cannabinoid formulations, and outcome measures limited direct comparison of the findings.

## Conclusions

This scoping review highlights the complex and variable role of cannabinoids in managing chemotherapy-related symptoms. Evidence supporting the use of cannabinoids, particularly nabilone, for reducing chemotherapy-induced vomiting was more consistent, although much of this evidence was derived from older studies using antiemetic regimens that differ from current standards of care. In contrast, evidence supporting improvements in appetite, weight gain, and other nutritional outcomes was limited and inconsistent, with most studies demonstrating no significant benefit compared with placebo or standard treatments. While a small number of pilot studies reported favorable findings, these require confirmation in larger, well-designed randomized controlled trials. Future research should also incorporate standardized nutritional outcome measures to better define the role of cannabinoids in supportive oncology care. Until then, clinicians should remain informed of emerging evidence and consider cannabinoid therapy within an individualized, patient-centered approach when clinically appropriate.

## Appendices

The Embase search was conducted on September 11, 2024, and yielded 232 results.

**Table 3:** Search queries for Embase

1	'chemotherapy'/exp	879302
2	chemotherapy*:ab,ti,kw OR 'cancer chemotherapy*':ab,ti,kw OR 'tumor chemotherap*':ab,ti,kw OR carcinochemotherap*:ab,ti,kw OR 'tumour chemotherap*':ab,ti,kw	779717
3	#1 OR #2	1181014
4	'cannabinoid'/exp	97057
5	cannabinoid*:ab,ti,kw OR anandamide:ab,ti,kw OR cannabichromene:ab,ti,kw OR cannabidiol:ab,ti,kw OR 'cannabidiol derivative':ab,ti,kw OR 'cannabidiol dimethyl ether':ab,ti,kw OR 'cannabidiol methyl ether':ab,ti,kw OR cannabidivarin:ab,ti,kw OR cannabielsoin:ab,ti,kw OR cannabigerol:ab,ti,kw OR 'cannabinoid derivative':ab,ti,kw OR cannabinol:ab,ti,kw OR 'cannabinol derivative':ab,ti,kw OR cannabis:ab,ti,kw OR 'cannabis derivative':ab,ti,kw OR deacetyllevonantradol:ab,ti,kw OR 'delta8 tetrahydrocannabinol':ab,ti,kw OR 'delta8 tetrahydrocannabinol derivative':ab,ti,kw OR 'delta9(11) tetrahydrocannabinol':ab,ti,kw OR dexanabinol:ab,ti,kw OR dextronantradol:ab,ti,kw OR dronabinol:ab,ti,kw OR ehp102:ab,ti,kw OR endocannabinoid:ab,ti,kw OR etrinabdione:ab,ti,kw OR lenabasum:ab,ti,kw OR levonantradol:ab,ti,kw OR 'medical cannabis':ab,ti,kw OR methanandamide:ab,ti,kw OR 'n oleoylethanolamine':ab,ti,kw OR nabiximols:ab,ti,kw OR nantradol:ab,ti,kw OR 'noladin ether':ab,ti,kw OR onternabez:ab,ti,kw OR palmidrol:ab,ti,kw OR suvadronabinol:ab,ti,kw OR tetrahydrocannabinol:ab,ti,kw OR 'tetrahydrocannabinol derivative':ab,ti,kw OR 'tetrahydrocannabinolic acid':ab,ti,kw OR virodhamine:ab,ti,kw	78622
6	#4 OR #5	114441
7	#3 AND #6	2480
8	'appetite'/exp	29601
9	'appetite':ab,ti,kw OR 'appetite regulation':ab,ti,kw OR 'food intake':ab,ti,kw OR 'food ingestion':ab,ti,kw OR 'food consumption':ab,ti,kw	143044
10	#8 OR #9	152553
11	'body weight gain'/exp	139673
12	'weight gain':ab,ti,kw OR 'body weight increase':ab,ti,kw OR 'body weight gain':ab,ti,kw OR 'weight increase':ab,ti,kw OR 'physiological well being':ab,ti,kw	114923
13	#11 OR #12	806416
14	#10 OR #13	936827
15	#7 AND #14	232

The Ovid MEDLINE search was conducted on September 11, 2024, and yielded 245 results.

**Table 4:** Search queries for Ovid MEDLINE

1	exp Cannabinoids/	19467
2	(Cannabinoid* or anandamide or cannabichromene or cannabidiol or "cannabidiol derivative" or "cannabidiol dimethyl ether" or "cannabidiol methyl ether" or cannabidivarin or cannabielsoin or cannabigerol or "cannabinoid derivative" or cannabinol or "cannabinol derivative" or cannabis or "cannabis derivative" or deacetyllevonantradol or "delta8 tetrahydrocannabinol" or "delta8 tetrahydrocannabinol derivative" or "delta9(11) tetrahydrocannabinol" or dexanabinol or dextronantradol or dronabinol or ehp102 or endocannabinoid or etrinabdione or lenabasum or levonantradol or "medical cannabis" or methanandamide or "n oleoylethanolamine" or nabiximols or nantradol or "noladin ether" or onternabez or palmidrol or suvadronabinol or tetrahydrocannabinol or "tetrahydrocannabinol derivative" or "tetrahydrocannabinolic acid" or virodhamine).ab,ti,kf.	58650
3	exp Chemotherapy/	1530867
4	(Chemotherapy* or 'cancer chemotherapy*' or 'tumor chemotherap*' or carcinochemotherap* or 'tumour chemotherapy*).ab,ti,kf.	475984
5	exp appetite/	11913
6	(appetite' or 'appetite regulation' or 'food intake' or 'food ingestion' or 'food consumption').ab,ti,kf.	101076
7	exp "weight gain"/	38032
8	('weight gain' or 'body weight increase' or 'body weight gain' or 'weight increase' or 'physiological well being').ab,ti,kf.	82136
9	1 or 2	60457
10	3 or 4	1849271
11	5 or 6	104197
12	7 or 8	97268
13	11 or 12	191779
14	9 and 10 and 13	245

The Web of Science search was conducted on September 11, 2024, and yielded 112 results.

**Table 5:** Search queries for Web of Science

1	TS=(Chemotherapy* OR ‘cancer chemotherapy*’ OR ‘tumor chemotherap*’ OR carcinochemotherap* OR ‘tumour chemotherapy*’)	627289
2	TS=(Cannabinoid* OR anandamide OR cannabichromene OR cannabidiol OR “cannabidiol derivative” OR “cannabidiol dimethyl ether” OR “cannabidiol methyl ether” OR cannabidivarin OR cannabielsoin OR cannabigerol OR “cannabinoid derivative” OR cannabinol OR “cannabinol derivative” OR cannabis OR “cannabis derivative” OR deacetyllevonantradol OR “delta8 tetrahydrocannabinol” OR “delta8 tetrahydrocannabinol derivative” OR “delta9(11) tetrahydrocannabinol” OR dexanabinol OR dextronantradol OR dronabinol OR ehp102 OR endocannabinoid OR etrinabdione OR lenabasum OR levonantradol OR “medical cannabis” OR methanandamide OR “n oleoylethanolamine” OR nabiximols OR nantradol OR “noladin ether” OR onternabez OR palmidrol OR suvadronabinol OR tetrahydrocannabinol OR “tetrahydrocannabinol derivative” OR “tetrahydrocannabinolic acid” OR virodhamine)	79540
3	TS=(“weight gain” OR “body weight increase” OR “weight increase” OR “body weight gain” OR “physiological well being")	119607
4	TS=("appetite" OR "appetite regulation" OR "food intake" OR "food ingestion" OR "food consumption")	139103
5	#3 OR #4	246,751
6	#1 AND #2 AND #5	112
